# The effects of a 6-week core exercises on swimming performance of national level swimmers

**DOI:** 10.1371/journal.pone.0227394

**Published:** 2020-08-31

**Authors:** Jakub Karpiński, Wojciech Rejdych, Dominika Brzozowska, Artur Gołaś, Wojciech Sadowski, Andrzej Szymon Swinarew, Alicja Stachura, Subir Gupta, Arkadiusz Stanula

**Affiliations:** 1 Institute of Sport Science, The Jerzy Kukuczka Academy of Physical Education, Katowice, Poland; 2 Faculty of Science and Technology, University of Silesia in Katowice, Katowice, Poland; 3 Faculty of Medical Sciences, University of West Indies, Cave Hill, Wanstead, Barbados; São Paulo State University (UNESP), BRAZIL

## Abstract

The aim of this study was to assess the impact of a 6-week specialized training program aimed at strengthening core muscles to improve the effectiveness of selected elements of a swimming race on a group of Polish swimmers. Sixteen male national level swimmers (21.6 ± 2.2 years) participated in the research. The competitors were randomly assigned to 1 of 2 groups before the data collection process: an experimental (EG, n = 8) and a control (CG, n = 8) group. Both groups of swimmers underwent the same training program in the water environment (volume and intensity), while swimmers from the EG additionally performed specific core muscle training. The task of the swimmers was an individual front crawl swim of 50 m, during which the kinematic parameters of the start jump, turn and swimming techniques were recorded using a video camera system. In both groups, a minor increase in the flight phase was observed at the start (EG = 0.06 m, 1.8%; *p* = *0*.*088*; CG = 0.08 m, 2.7%; *p* = *0*.*013*). The time to cover a distance of 5 m after the turn and the recorded average speed in swimming this distance for the EG statistically significantly improved by 0.1 s (-28.6%; *p* < *0*.*001*) and 3.56 m∙s^-1^ (23.2%; *p* = *0*.*001*), respectively. In the EG, a statistically significant improvement in 50 m front crawl swimming performance of 0.3 s (-1.2%, *p* = *0*.*001*) was observed. The results of the research show that the implementation of isolated strengthening of the stabilizing muscles seems to be a valuable addition to the standard training of swimmers.

## Introduction

Strength and muscular power are significant determinants of success in swimming-related sports. Appropriate training of the abdominal muscles and torso seems to be one of the key elements determining the effectiveness of the training process [1]. The main goal of swimming competition is to overcome the given distance in the shortest possible time, which is achieved mainly by proper body positioning in the water and minimizing resistance [2–5]. Numerous publications show that exercises strengthening the core muscles are an integral part of many swimming training programs [4,6,7]. Increased work of the stabilizing muscles can form the basis for generating more strength through the limbs [8,9]. According to many sources, the concept of core muscles is expanding, including the rectus abdomen, latissimus dorsi, gluteus maximus or trapezius [6,10]. Proper control of the body position while swimming at a distance, as well as during the start jump and turn, increases the efficiency and thus reduces the distance traveled [11].

Appropriate strengthening of the muscles responsible for the correct positioning of the body is fundamental to the swimming technique [12]. This involves correct positioning of individual body segments, i.e., the head, shoulder girdle, torso, pelvis girdle and legs. Efficiency in swimming can be achieved if these muscles follow a nearly linear arrangement, thereby minimizing the resistance applied by the water to the body [11,13,14]. The unstable background against which the body of the swimmer is located requires exemplary core muscle work, and a lack of stable support implies a deficit of one or several muscles, which can cause significant time losses. In addition to minimizing resistance, an appropriate high and stable body position allows one to optimize the power of his or her upper and lower limbs [4,15,16].

There is much evidence in the literature on the effectiveness of dry-land training in improving the results achieved in swimming [17,18]. In the research of Patil et al. [4], in accordance with the authors' expectations, the proposed specialized core muscle strengthening training improved the performance of this area (functional core muscle strength test) and led to significant improvement during a 50 m front crawl swim.

Additionally, Gencer’s [19] experiment aimed to review the effects of an 8-week core training program to see how certain physical and motor attributes change, as well as measure the difference in performance in front crawl swimming by female athletes. The results showed that the experimental group significantly improved their performance in the 50-meter front crawl time trial. The authors also noted an improvement in horizontal jump, vertical jump, and push-ups after the prescribed 8-week training block. Similar conclusions were drawn by Gönener et al. [20], who stated that training with the use of Thera-Band tapes (including their use to engage core muscles) improves the performance of swimmers. Core muscle training has been widely researched in the recent years, and even though it seems like there is an universal conviction that the correct strength of stabilizing muscles improves the athletic level of competitors, some papers show only marginal impact of this type of training on the final sporting success [7,10,19]. Many studies do not show a direct relationship between improving muscular strength on land and improving the results achieved by swimmers in the water [21,22]. Inconsistent findings of the previous research encouraged us to take a different approach and study the effects of dry-land training (with emphasis on strengthening core muscles) on selected kinematic parameters and performance in 50 m front crawl swimming.

We hypothesize that strengthening the core muscles will positively impact the effectiveness of the studied elements of the swimming start, turn technique and other aspects of a swimming race over a distance of 50 m. We also assume that as a result of the experiment, this strengthening will improve the completion time for a 50 m front crawl swim.

Thus, the present study aimed to investigate the effect of strengthening core muscles as a result of dry-land training in a 50 m front crawl performance of national level male swimmers. This study will also examine the effect of strengthening core muscles on a number of kinematic variables in front crawl swimming.

## Methods

### Participants

Sixteen male national level swimmers who are members of the Polish National Swimming Team (seniors and youth) were involved in this research. The competitors had a minimum of 10 years of training experience, and their best results were at least 800 points according to the FINA classification. The participants in the experiment were in the same period of preparation for competition, i.e., in the subperiod of specific preparation. The competitors were randomly placed into either an experimental group (EG) of 8 swimmers or a control group (CG) of 8 swimmers. Both groups of swimmers carried out the same training program (10 training sessions in water and 2 training sessions in the gym per week), while swimmers in the EG additionally carried out specialized core muscle training (SCMT), which took place 3 times a week for 6 weeks. For both groups, water training took place from 6:00–8:00 a.m. and 5:00–7:00 p.m., and the strength training occurred on the days when the swimmers did not perform SCMT. Both strength training and experimental training took place after the water training. The training performed in the experiment did not disturb the preparation of the swimmers to start in competitions. All participants had up-to-date medical examinations, and any contraindications to participate in the studies were excluded. None of the swimmers were taking drugs, medication, or dietary supplements known to influence physical performance. During the experiment, the subjects were tested on an equal and balanced diet. The calorific value was selected individually based on the measurement of body mass composition and the volume and intensity of the training program. The swimmers also offered written consent to participate in the experiment. All the swimmers participating in this study were informed about the procedures, goals, and expected duration of the experiment. They were also informed that they were free to withdraw from the research at any stage. The research project was approved by the University Bioethics Committee for Research at The Jerzy Kukuczka Academy of Physical Education in Katowice (No. 8/2018). Anthropometric data of the competitors are presented in Table 1. Body height was assessed using a stadiometer (Seca 213, Seca GmbH & Co, Hamburg, Germany) with a precision of 0.5 cm, while the body mass and its composition were determined by the method of electrical impedance using the InBody 220 device (Biospace Co. Japan).

**Table 1:** Physical characteristics of participants (mean ± SD).

Variable	Experimental group (n = 8)	Control group (n = 8)	*p-value*
Age (year)	20.2 ± 1.17	20.0 ± 1.9	0.606
Body mass (kg)	74.9 ± 10.67	75.4± 6.27	0.926
Height (cm)	183.0 ± 6.57	182.1 ± 3.18	0.761
Fat mass (%)	6.52 ± 3.22	8.09 ± 2.23	0.140
Lean body mass (kg)	30.5 ± 5.46	29.4 ± 1.31	0.101
Fat mass of trunk (%)	5.75 ± 3.01	7.96 ± 2.30	0.124

### Procedures

The training program, which lasted six weeks, consisted of 18 units of targeted dry-land training. The duration of the main unit did not exceed 25 minutes. According to the purpose of the research, the developed training program included exercises involving the core muscles. In the general sense, we referred to them as torso muscles or, less recently, used the term "body core". Comparing with other definitions of this term, we find a common denominator, i.e., the deep muscles that provide stabilization of the whole body and the basis for functional stability of the lumbar, sacral and iliac areas [6,10,12,14]. SCMT consists of four exercises: flutter kicks (scissors), single leg V-ups, prone physio ball trunk extension, and Russian twists. Progression consisted of changing the position of the body, adding a motion element, adding an unstable ground and increasing the resistance. The same training units were carried out three times a week. Depending on the exercise, the level of difficulty progressed in weekly or biweekly cycles. If a swimmer was unable to complete the task with a certain resistance, he returned to the load from the previous microcycle until the end of the duration of the given exercise. All exercises were performed in 4 series, with a 40-second work schedule and a 20-second break between sets. The duration of the training and the number of series were based on the coaching experience of the authors, but they are also justified by the literature. Many authors [11,23] suggest a temporary dosage of exercises in core muscle training and a certain number of series. Based on these, our research protocol was established. The details of the training program are presented in Table 2.

**Table 2:** A brief description of the exercises of SCMT and their progression over a 6-week training program.

Week of training	Flutter kicks (scissors)	Single leg V-ups	Prone physio ball trunk extension	Russian twists
1	Arms crossed on the chest	No extra load	Arms crossed on the chest	No extra load
2	Streamlined position	No extra load	Arms crossed on the chest	No extra load
3	Arms crossed on the chest + weights on the ankles	Dumbbells in hands	Holding medicine ball	Holding kettlebell
4	Streamlined position + weights on the ankles	Dumbbells in hands	Holding medicine ball	Holding kettlebell
5	Arms crossed on the chest + weights on the ankles (swimmer performs this progression on a wiggle cushion)	Dumbbells in hands + weights on the ankles	Medicine ball trunk extension throw	Holding kettlebell (swimmer performs this progression while sitting on a wiggle cushion)
6	Streamlined position + weights on the ankles (swimmer performs this progression on a wiggle cushion)	Dumbbells in hands + weights on the ankles	Medicine ball trunk extension throw	Holding kettlebell (swimmer performs this progression while sitting on a wiggle cushion)

The tests consisted of two stages: one preceding the experiment and one performed after the experiment. During the research, the same procedure was carried out at the same time of day and with the same order of athletes. The measurements were carried out in a 25 m swimming pool (The Jerzy Kukuczka Academy of Physical Education in Katowice) three days before and after the core muscle training was completed. During the tests, the air temperature was ~25°C, the water temperature was ~27°C, the water pH was ~6.93, and the relative air humidity was ~60%. The task of the swimmers was to swim 50 m front crawl technique from the starting block under race conditions. To accurately measure the times achieved by the participants, the Omega electronic time measurement system was used (OMEGA S.A., Switzerland). The swimming race was recorded using two digital video cameras (JVC GC-PX100BE, Japan) with a rapid shutter speed (1/1000 s) operating at a sampling rate of 50 Hz. One of the cameras was set 1.5 m above the water at a distance of 2 m from the starting wall perpendicular to the direction of the road traveled by the swimmer to register the dive start and the entrance of the swimmer into the water. The second camera was placed 1.5 m above the water exactly in the middle of the swimming pool lengthwise (12.5 m from the starting wall) to capture the distance swum. Both cameras were mounted on tripods positioned at poolside 0.5 m from the edge of the pool perpendicular to lane 2. To register the glide after the turn, a third camera (Sony FDR-X3000, Japan) was placed underwater at a distance of 2 m from the turning wall at a depth of 1.0 m at the sidewall of the pool basin; the lens of this camera covered both the turning wall and a mark located 5 m from the turning wall. These cameras were calibrated using a series of poles of known lengths positioned at specifically known positions throughout the length of the area that the swimmers traveled during each trial. The following parameters of the dive start were analyzed: entry distance (cm), time in the air with take-off (s), reaction time (s), time in the air (s), entry velocity (m∙s^-1^), and dive angle (°). The time was measured when a swimmer reached a distance of 5 m after the turn, and then the speed of swimmer after the turn after completion of the first 5 m was calculated. Additionally, based on the swimming velocity data and the duration of three complete stroke cycles, the stroke rate (SR) (cycles∙s^-1^) and the stroke length (SL) (m) were determined (a detailed description of all measured parameters is provided in Table 3). All video files were analyzed by 2 different researchers with experience in digitization management via the Kinovea software (v. 0.8.26, Kinovea, Paris, France), which allowed time-motion analysis of the registered elements. To assess the reliability of the digitizing process (interobserver), 6 trials were quantified using intraclass correlation coefficients (ICCs). The ICCs ranged from 0.979 (95% CI, 0.972–0.984) to 0.994 (95% CI, 0.983–0.997).

**Table 3:** Detailed description of the parameters measured by using the Kinovea software while swimming 50 m front crawl.

Entry distance (cm)	Distance from the starting wall to the head entry point. It is considered as the length of the flight and is measured parallel (horizontal) to the water surface.
Entry velocity (m∙s^-1^)	The horizontal velocity of the swimmer traveling through the air during the flight phase before entry into the water (based on the length of the flight phase and the time in the air).
Time in the air with take-off (s)	This is the sum of the “flight phase” and the “reaction time”.
Time in the air (s) (flight phase)	The time from when the swimmer leaves the block to when the swimmer’s head enters the water. It is also known as the flight phase.
Dive angle (degrees)	The angle at which the swimmer enters the water. It is the angle between the water surface and the central axis of the body at the time when the head touches the water surface.
Reaction time (s)	The time needed by the swimmer to leave the block following the starting signal. It is considered as the reaction time.
Time 5 m after the flip turn (s)	The time needed by the swimmer to reach the 5 m line after the turn. It covers the period between when the swimmer pushes off the wall and when the swimmer’s head crosses the 5 m line.
Average velocity after the flip turn (m∙s^-1^)	The horizontal velocity, which the swimmer reaches 5 m after pushing off the wall.
Swimming velocity (m∙s^-1^)	The horizontal velocity, which the swimmer obtains after swimming a distance of 5 m. It was measured between 12.5 and 17.5 m during the first and second 25 m.
Duration of 3 cycles (s)	The time needed by the swimmer to perform 3 strokes. It was measured for the first and second 25 m.
Stroke rate (cycles∙s^-1^)	The time required to perform 3 stroke cycles was measured (in the middle section of the first and the second lap) and then used to calculate the stroke rate; SR = 60 × 3/tSR (SR: stroke rate, tSR: duration of 3 cycles).
Stroke length (m)	The distance covered in one stroke. It was calculated by dividing the swimmer’s distance by the stroke rate. The SL calculation was based on the data gathered in 9 m sectors of the 50 m distance during both laps (for the first lap, between 15 and 24 m, and for the second lap, between 40 and 49 m).
Total time to complete the 50 m (s)	The total time needed to cover the distance of 50 m from the starting signal until the wall is touched by hand of the swimmer at the end.

### Statistical analysis

Means and standard deviations were used to represent the average and typical spread of values of all performance variables of the swimmers. The normal Gaussian distribution of the data was verified by the Shapiro-Wilk’s test. Levene's test for the equality of means showed no significant differences in the group variances. A two-way analysis of variance with repeated measures and a Bonferroni post hoc test were used to investigate the main effects and the interaction between the group factor (experimental vs. control) and time factor (pretraining vs. post-training), as well as the existence of differences between groups in the initial and final data of all variables.

The magnitudes of the differences between the results of the pretest and posttest were expressed as relative differences in percentages and as standardized mean differences (Cohen effect sizes). The criteria to interpret the magnitude of the effect sizes were as follows: <0.2, trivial; 0.2–0.6, small; 0.6–1.2, moderate; 1.2–2.0, large; and >2.0, very large. Additionally, the absolute and percentage change from pre- to post-test was calculated for all variables for each group.

Statistical power equations were used to determine the minimum study population at the *p < 0*.*05* level with a power of 0.8 and revealed a sample of a minimum of 6 subjects in each group. Statistical significance was set at *p < 0*.*05*. All statistical analysis were conducted using Statistica 13.3 (TIBCO Software Inc.).

## Results

Table 4 shows the results of all measurements before (pretest) and after (posttest) training.

**Table 4:** Pre- and post-training values of the performance variables for swimmers. In each data block, the upper row is for the EG, and the lower row is for the CG.

Performance variable	Pretraining Mean ± SD	Post-training Mean ± SD	Change Δ (%) [±95% CI]	*p*	ES / rating	ANOVA (F, *p*)					
						Time effect		Group effect		Time × Group	
						F	*p*	F	*p*	F	*p*
Entry distance (m)	3.11 ± 0.09	3.16 ± 0.08	0.06 (1.8%) [-0.01; 0.13]	.088	0.66 / Moderate	13.39	**.003**	6.75	**.021**	0.39	.545
	2.96 ± 0.13	3.04 ± 0.12	0.08 (2.7%) [0.02; 0.14]	**.013**	0.65 / Moderate						
Entry velocity (m∙s^-1^)	12.77 ± 1.65	13.34 ± 1.47	0.57 (4.3%) [0.11; 1.02]	**.021**	0.36 / Small	0.03	.860	0.41	.533	3.01	.105
	13.99 ± 2.87	13.53 ± 2.81	-0.46 (-3.4%) [-1.79; 0.87]	.438	0.16 / Trivial						
Time in the air with take-off (s)	1.05 ± 0.03	0.95 ± 0.05	-0.09 (-9.7%) [-0.13; -0.06]	**< .001**	2.14 / V. large	34.91	**< .001**	1.48	.243	10.24	**.006**
	1.05 ± 0.10	1.03 ± 0.08	-0.03 (-2.7%) [-0.06; 0.01]	.092	0.32 / Small						
Time in air (s)	0.25 ± 0.04	0.24 ± 0.03	-0.01 (-3.1%) [-0.02; 0.01]	.285	0.22 / Small	0.04	.846	0.76	.397	1.92	.188
	0.22 ± 0.05	0.23 ± 0.06	0.01 (4.4%) [-0.02; 0.04]	.388	0.19 / Trivial						
Dive angle (^0^)	40.13 ± 4.36	39.75 ± 4.23	-0.38 (-0.9%) [-3.53; 2.78]	.787	0.09 / Trivial	0.01	.929	1.76	.206	0.41	.535
	37.38 ± 3.25	37.88 ± 2.95	0.50 (1.3%) [-0.27; 1.27]	.170	0.16 / Trivial						
Reaction time (s)	0.80 ± 0.03	0.71 ± 0.03	-0.09 (-11.9%) [-0.12; -0.05]	**.001**	2.87 / V. large	33.73	**< .001**	11.53	**.004**	2.70	.123
	0.83 ± 0.05	0.79 ± 0.04	-0.05 (-6.1%) [-0.09; -0.01]	**.025**	1.02 / Moderate						
Time 5 m after the turn (s)	0.43 ± 0.06	0.34 ± 0.06	-0.10 (-28.6%) [-0.12; -0.07]	**< .001**	1.51 / Large	41.10	**< .001**	4.98	**.043**	1.83	.194
	0.50 ± 0.11	0.44 ± 0.08	-0.06 (-14.2%) [-0.11; -0.01]	**.026**	0.65 / Moderate						
Average velocity 5 m after the turn (m∙s^-1^)	11.77 ± 1.68	15.34 ± 2.80	3.56 (23.2%) [2.16; 4.97]	**.001**	1.54 / Large	39.58	**< .001**	6.13	**.027**	9.55	**.008**
	10.37 ± 2.14	11.58 ± 2.11	1.22 (10.5%) [0.1; 2.33]	**.037**	0.57 / Small						
Stroke rate (cycles∙s^-1^)	1.02 ± 0.08	1.03 ± 0.08	0.02 (1.5%) [-0.01; 0.04]	.242	0.19 / Trivial	1.80	.201	3.36	.088	0.63	.441
	0.97 ± 0.04	0.97 ± 0.05	0.00 (0.4%) [-0.01; 0.02]	.633	0.09 / Trivial						
Stroke length (m)	1.63 ± 0.15	1.58 ± 0.16	-0.05 (-3.5%) [-0.12; 0.01]	.091	0.36 / Small	3.50	.083	0.06	.805	3.24	.094
	1.59 ± 0.06	1.59 ± 0.08	0.00 (-0.1%) [-0.03; 0.02]	.924	Trivial						
Total time to cover 50 m (s)	25.24 ± 0.35	24.94 ± 0.49	-0.3 (-1.2%) [-0.43; -0.16]	**.001**	0.71 / Moderate	8.89	**.010**	15.13	**.002**	0.58	.458
	26.82 ± 1.09	26.64 ± 1.19	-0.18 (-0.7%) [-0.53; 0.18]	.274	0.16 / Trivial						

CI–confidence interval; ES–effect size: <0.2, trivial; 0.2–0.6, small; 0.6–1.2, moderate; 1.2–2.0, large; >2.0, very large; Δ (%)–Absolute and percentage of change from pre- to posttest; *p*–*p*-value.

In both the EG and CG, after the end of the 6-week training, we could observe an increase in the entry distance during take-off. In the EG, the improvement was 0.06 m (1.8%, *p = 0*.*088*, ES = *Moderate*), while in the CG, the improvement was 0.08 m (2.7%, *p = 0*.*013*, ES = *Moderate*). With the elongation of the flight phase in the EG, a statistically significant increase in the entry velocity was noted at 0.57 m∙s^-1^ (4.3%, *p = 0*.*021*, ES = *Small*), accompanied by a statistically significant reduction in the time in the air with take-off by 0.09 s (−9.7%, *p < 0*.*001*, ES = *Very large*). ANOVA revealed a significant interaction (training group × test time point) for the time in the air with take-off (*F*_(1,14)_ = 10.242, *p = 0*.*006*). In both the EG and CG, a statistically significant reduction in reaction time on the starting platform of 0.09 s (−11.9%, *p = 0*.*001*, ES = *Very large*) and 0.05 s (−6.1%, *p = 0*.*025*, ES = *Moderate*), respectively, was recorded.

At the end of the experiment, the time after covering a distance of 5 m after the turn and the recorded average speed in swimming this distance in both the EG and CG improved. In the EG, the abovementioned elements of the swimming race were significantly improved by 0.1 s (−28.6%, *p < 0*.*001*, ES = *Large*) and 3.56 m∙s^-1^ (23.2%, *p = 0*.*001*, ES = *Large*), respectively, while in the CG, these parameters improved by 0.06 s (−14.2%, *p = 0*.*026*, ES = *Moderate*) and 1.22 m∙s^-1^ (10.5%, *p = 0*.*037*, ES = *Small*), respectively. ANOVA revealed a significant interaction for the average velocity 5 m after the turn (*F*_(1,14)_ = 9.547, *p = 0*.*008*).

The result of all observed changes was the value of the last of the tested parameters–the time required to cover the distance of 50 m via front crawl swimming. In the EG, a statistically significant improvement in athletic performance of 0.3 s (−1.2%, *p = 0*.*001*, ES = *Moderate*) was observed, while swimmers in the CG had a statistically insignificant improvement in athletic performance of 0.18 s (−0.7%, *p = 0*.*274*, ES = *Trivial*).

## Discussion

In this article, it was hypothesized that in a selected group of swimmers of either senior or adolescent age, strengthening of the core muscles will positively impact the effectiveness of the studied elements of a 50 m swimming race, which may lead to an improvement in sports results.

Both in the EG and CG, the parameter of the entry distance improved, which may indicate a positive aspect of the training carried out by the competitors in a given period. Notably, the specialized core muscle training did not change the value of this parameter, further reducing the start jump time (parameter improvement of 0.09 s, ES = *Very large*), which is the result of the reaction time and flight phase time measured until the swimmer touches the water surface with the head. The value of this parameter among EG athletes was statistically significantly lower (*p* < *0*.*001*), while the improvement that followed was higher than in the CG. In the EG, there was a statistically significant increase in the speed of entry of the swimmer into the water (4.3%, ES = *Small*), in contrast to the CG, in which regression of the analyzed velocity (−3.4%) occurred.

As published research results show, the start jump in swimming directly affects the competitive level, depending on the type of competition, and especially the distance covered, as it accounts for 0.8% of the time needed to complete 1500 m and 26.1% of the time required to complete 50 m (front crawl) [24,25]. According to the assumptions of this experiment, one of the analyzed parameters was the start jump, which can be divided into three stages: on the starting block, flight and the underwater phase [25]. In this work, the first two were analyzed, and it is worth noting that under the influence of SCMT, the studied swimmers improved their reaction times. However, these studies did not measure swimmers’ reaction time (the time taken by the swimmers to leave the starting block following starting signal) [26,27], which is a neuromuscular skill playing a very important role especially in short distance swimming. Specialized core muscle training in this study significantly (*p = 0*.*001*, ES = *Very large*) improved the reaction time of the swimmers.

These results are consistent with the work of Rejman et al. [26], in which the time on the starting block was shortened due to a six-week plyometric training, and the speed of a swimmer achieved during the flight phase increased (0.71 m∙s^-1^), which may be related to an improvement in lower limb power [26]. Although there was no statistically significant increase in swimmer entry velocity into the water, in the EG, there was an improvement in this parameter, in contrast to the CG, in which there was a statistically insignificant regression of the analyzed velocity. It seems that under the influence of core muscle training, the integration of the muscles of the lower and upper limbs and torso improved, which translated into a more efficient transfer of energy from the lower limbs to the body and further to the arms and thus a more efficient (faster) torpedo (starting) position [8,15].

Many studies show the importance of the flight phase, the maximization of which, combined with the appropriate entry into the water, allows a swimmer to achieve higher speeds during the underwater phase [28,29]. The distance of the flight phase is a very important parameter of the effectiveness of a swimmer during a race because the body travels much faster in the air than in the water [30]. In the study of Breed and Young [24], dry-land resistance training did not affect the distance of the flight phase during the starting jump, which may be related to its specificity. In the studies conducted by the authors, in both the EG and CG, there was a statistically significant improvement in the length of the flight phase parameter, which may indicate a positive aspect of the training carried out by the swimmers in the given start-up preparation period. Notably, specialized core muscle training did not affect the value of this parameter and shortened the start-up time, which is the resultant of the reaction time and flight phase time measured until the swimmer touches the water surface with the head. The glide speed after the start jump is highly dependent on the time of entry into the water, swimmers position, direction and depth of entry [31,32]. In studies based on a correlation analysis, it was determined that there is a strong relationship between "take-off horizontal velocity and time on block" and the time obtained by competitors after an initial distance of 15 meters [28,33]. Increasing the take-off horizontal velocity should cause the swimmer to enter the water at a smaller angle. In the conducted studies, in the EG group, an improvement in the flight phase velocity and a decrease in the swimmer entry angle (statistically insignificant) were observed, while in the CG group, both parameters did not improve. According to other studies, it can be presumed that an incorrect position upon entry into the water, despite the appropriate speed of the starting jump, will not translate into the speed that the swimmer will reach during the underwater phase [27].

In both analyzed groups, a decrease in swimming time in the first 5 m after the turn was observed, and the decrease in this value was statistically significant in the EG (an improvement of 28.6%, *p < 0*.*001*, ES = *Large*). Moreover, it significantly influenced the next analyzed parameter, i.e., the speed of the swimmer 5 m after the return from the turn wall; this value improved by 23.2% (*p = 0*.*001*, ES = *Large*). There are very few studies in the literature investigating the effectiveness of swimming turns, especially the tumble turns, due to a lack of appropriate technologies and other factors. The swimming turn is a complicated technical element due to the environment in which it takes place, multilevel and multiaxis movement, and the number of involved body segments [34]. It is undeniable that a properly performed turn can improve the total swimming duration [34]. It is known that a slight improvement in the components of the turn can improve the effectiveness of swimming over the total distance. One of the elements of the turn is the glide, which may depend on the push-off and the proper position of the swimmer's body [4,11]. Seemingly, the decrease in the time required to cross the first few meters after the turn may significantly affect the final time measured at the end of the race. In the EG, an increase in the SR of 1.5% was observed (ES = *Trivial*), as well as shortening of the swimming SL, which, for competitors performing core muscle training, decreased by 3.5% (*p = 0*.*091*, ES = *Small*). There were no significant changes among CG competitors. The increase in the speed of swimming may be caused by an increase in stroke length with a simultaneous drop in the stroke rate, but it can also be achieved only by extending the swim stroke length [35]. In the work of Patil et al. [4], there were no statistically significant changes in the stroke rate or stroke length under the influence of core muscle training. The lack of similar results of various studies can be explained by another preparatory period in which the experiments were carried out. In addition, many authors have determined the SR and SL to be factors of swimming performance, which is associated mainly with strength and muscular power [36]. The competition over the mentioned distances researched by the authors for the needs of this study is characterized by high dynamics. The desired effect of directed stabilization muscle training will seemingly be observed at longer distances, e.g., 200 m, where the correct position of the swimmer's body seems to be crucial, and thus, the stroke length may be longer.

The result of all the observed changes was the value of the last tested parameter–the time required to cover a distance of 50 m. In the EG, a statistically significant improvement in athletic performance of 1.2% (*p = 0*.*001*, ES = *Moderate*) was observed. During the final test, swimmers in the CG also achieved a better result, but this improvement was not statistically significant. On the basis of the available literature, a rational explanation for this issue may be the increased core muscle activity, which allows for a more effective transfer of strength between the limbs and for maintaining the body in a streamlined position [11,37]. There are many papers on the impact of dry-land training on performance in swimming sports; however, the results of these studies are not consistent. For example, Tanaka et al. [37] suggest that the increase in strength achieved through resistance land training does not affect the swimmer's driving force in the water and therefore does not improve swimming performance [37–39]. A large improvement was observed in the study of Weston et al. [10]; however, it may be caused by the much longer period of the specialized training program, as well as the younger research group. Another study found an improvement in central stabilization that did not translate into swimming efficiency [4]. However, there are numerous studies proving the positive impact of dry-land training on the results of swimming, and the recorded progression of results oscillates between 1.3% and 4.4% [12,40]. The results obtained by the authors of the above studies are similar to the results of the work of Weston et al. [10], in which, as a result of twelve weeks of training involving core muscles, a 2% improvement in sprinting distance was observed. Patil et al. [4] also noted a statistical progression of the results achieved in competition after a six-week training intended to strengthen the stabilizing muscles. In this study, the improvement in the efficiency of individual swimming elements translated into better final competition results, i.e., shorter times required to cover a distance of 50 m. In the present study, it is likely that SCMT causes improvement of a number of swimming variables, which together result an overall increase in 50 m front crawl swimming performance by 1.2%, whereas the CG swimmers improved their performance just by 0.7%.

## Conclusions

The present study involved a group of selected swimmers who completed a specially designed training program aimed at improving the strength and endurance of their core muscles. The research results suggest that the implementation of isolated training to strengthen the stabilizing muscles seems to be a valuable addition to a standard swimming training. Based on the conducted experiment, it can be concluded that the described training affects the efficiency of swimming over a short distance. It is especially notable that in this study, the improvement in the efficiency of individual swimming elements did translate into better final sports results, i.e., shorter times required to swim a distance of 50 m. The authors observed a statistically significant progression of the results, which seems to be fundamental for the sprinting distance. In direct sports competition, even a slight improvement in time may guarantee final success. The novelty of this work is a detailed analysis of many parameters related to the techniques of swimming, including the start jump and turn. However, the similarity between the results of this experiment and those of other experiments indicates the need to continue research in the field of dry-land training for swimmers, especially the need to strengthen the core muscles. Future experiments should also be enriched with EMG tests showing proper and conscious tensioning of the stabilizing muscles.
